# The Role of Patient Organisations—Patients’ and Parents’ Views and Experience of Hirschsprung’s Disease

**DOI:** 10.3390/children11081006

**Published:** 2024-08-16

**Authors:** Sabine Alexander, Annette Lemli

**Affiliations:** SoMA e.V. German Patient Organisation for Anorectal Malformations and Hirschsprung’s Disease, 81825 Munich, Germany; annette.lemli@soma-ev.de

**Keywords:** Hirschsprung’s disease, patient associations/organisations, patient support group, self-help group, transition care

## Abstract

In many countries, patient organisations offer advice and the exchange of experiences to Hirschsprung’s disease patients and their families. Professional treatment by experienced health care providers and the availability of life-long multidisciplinary follow-up care are essential. However, outside the clinic, patients and their families have to manage life on a day-to-day basis at home, which often brings up uncertainties and questions: Parents go through different stages during the diagnosis and treatment of their child, the affected children themselves go through many different stages of development, and even through the course of adulthood, new questions regarding the chronic disease may arise. Patient organisations can support the patients and their families at all stages of life by listening, offering information in an understandable way, connecting people, and sharing others’ experiences. This enables families and patients to develop a better understanding of the rare disease and promotes their management strategies and confidence. The holistic approach of patient organisations aims to complement the medical treatment. Therefore, the referral of all patients and their families to patient organisations should be part of the medical advice in the treatment of Hirschsprung’s disease.

## 1. Introduction

In addition to the medical steps that have to be taken in the diagnosis and treatment of Hirschsprung’s disease, patient organisations can meet the needs of patients and their families outside the hospital in their day-to-day life. Patient organisations offer information in an understandable way and give advice on available support. They communicate their expertise gained from personal and collective experience. This enables patients and parents to gain a better understanding of the rare and complex condition so they can develop competence and confidence in managing day-to-day life with the chronic disease.

Patient organisations work differently in different countries: some are more active on social media and use networking online, while others also offer information, advice and the exchange of experiences in face-to-face meetings [1]. However, regardless of how the organisations are set up, the most important message that all patient organisations deliver is “You’re not alone in this!” [1,2].

In this article, the work of SoMA e.V., the German “patient organisation for people with anorectal malformations”, which also represents Hirschsprung’s disease, is presented. According to the association’s charter, SoMA e.V. aims to provide information about anorectal malformations and Hirschsprung’s disease and to support and give advice to patients and their families regarding all related issues. This is achieved by facilitating opportunities for information and experience exchange among patients, their families, clinicians and multidisciplinary experts. Furthermore, scientific research is promoted [2].

## 2. Patient Treatment

### 2.1. Diagnosis

The arrival of a new baby in a family is a major and critical life event, whether it is a family’s first child or whether there are already siblings waiting for their new brother or sister. As there is no prenatal diagnosis for Hirschsprung’s disease, in most cases, parents are expecting to welcome a healthy child into the world, and their baby looks healthy at birth [1,2,3,4,5,6]. When their child then presents with symptoms of Hirschsprung’s disease shortly after birth, the suspicion that the baby might be affected by a serious disease comes as a shock to the unprepared parents at a time where they are emotionally most vulnerable [3]. Parents then often find themselves in an overwhelming situation where they are suddenly confronted with a lot of medical information and potentially have to make difficult decisions about their child’s further treatment [4,5,6,7].

There are babies who do not present with symptoms straight away but develop them gradually, for example in the course of the introduction of solid food. This often means that the path to diagnosis is extended, and parents may have to live over a longer period with a great amount of uncertainty [3,8]. Knowing that something is wrong with their baby’s health but not yet knowing how to help their child puts an enormous psychological strain on the parents. Whilst managing day-to-day life with their unwell baby and potential siblings, they have to find the appropriate medical experts [4], which can be especially difficult in countries where the treatment of Hirschsprung’s disease is not centralized [9].

Naturally, in a hospital setting, the focus lies in medical treatment. For the parents, however, a lot more questions and uncertainties arise around the diagnosis [7]:

- What is essential to know about the nature and treatment of Hirschsprung’s disease?
- What support is available regarding medical care for our child before corrective surgery?
- Where do we find social, financial or psychological advice, if needed?
- How are we going to cope as a family?
- What does the diagnosis “Hirschsprung’s disease” mean for our child regarding their long-term quality of life?

Many patient organisations provide information like guidelines, patient journeys and general information in understandable language. Talking from the point of view of being parents of children with Hirschsprung’s disease themselves, they can reassure and encourage parents to whom the diagnosis is completely new. The parents gain a better understanding of the condition and are therefore able to support their child better in the medical process [10].

### 2.2. Surgery

Although parents know that surgery is needed to improve their baby’s health long-term, naturally, they have a lot of concerns regarding the complex corrective surgery:

- Have we chosen the right centre with enough expertise in the treatment of Hirschsprung’s disease?
- What complications might occur?
- What is to be expected after the pull-through operation?
- Will the treatment have psychological effects on our child?
- How can we best support our child?

In the family’s day-to-day life, there are probably no other parents who face a similar stressful situation and have an understanding of what the parents are going through at this stage. Patient organisations can fill this gap by connecting affected families and sharing their experiences [1,2]. For parents, surgery is a psychologically demanding process that requires emotional and medical support [8].

### 2.3. Follow-Up Care

When corrective surgery has been completed, parents are charged with managing day-to-day life at home, which is where questions arise over the course of time:

- How do we deal with residual symptoms?
- What multidisciplinary follow-up care is available?
- What is advisable regarding nutrition?
- How can we support our child at nursery and in school?
- What has helped other Hirschsprung’s disease children in similar situations?

In order to help the parents find answers to these questions, the German patient organisation “SoMA e.V.” runs regular seminars with multidisciplinary experts, like paediatric surgeons, nutritionists, specialist nurses, psychologists and physiotherapists [2]. During the online or face-to-face meetings, the experts provide basic information on different aspects of follow-up care and offer the possibility for patients and parents to ask questions and exchange experiences [2].

Quotes from parents after participating in SoMA seminars on various aspects of Hirschsprung’s disease:

“*We are relatively new to the subject and for us it was just great to hear about other people’s experiences and everyday tips*”.

“*I found it very informative, and above all, I gained new confidence regarding my son’s future*”.

“*It was especially interesting to hear about the experiences of other parents with older children*”.

“*The complex subject of genetics was explained in an understandable way. I found it very good though, that the seminar was not just about the medical side of things, but there was also room for psychological aspects*”.

“*Thank you very much for the interesting talk on nutrition. I found it a very encouraging tip to allow diversity in my nutrition*”.

## 3. Family Life

Family life with a child affected by a chronic disease comes with special demands regarding parenting: a child with Hirschsprung’s disease may need more nursing than healthy children, and there may be repeated hospital admissions and traumatic experiences of medical treatment. There may also be siblings in the family who need adequate attention from their parents as well [4,5,8].

- How do we handle difficult situations when our child is not complying with medical requirements?
- How can we best support our children emotionally?
- How can we do justice to the needs of siblings?
- How can we receive support for ourselves as parents?

SoMA e.V. offers workshops for parents to learn more about helpful parenting techniques and to offer emotional support for both parents and children. These online workshops are run by a qualified trainer for the “Hand-In-Hand” parenting concept [2].

There is also the offer of a SoMA e.V.-organised family holiday, as well as a gathering just for mums and one just for dads; these offer a short but relaxing time away from family responsibilities and the possibility to talk to other parents about the challenges of managing partnership and family life with a chronically ill child [2,4].

Quotes from parents after a parenting seminar:

“*It’s a great relief to be able to say things that you can’t normally share with the people around you*”.

“*I gained some important food for thought regarding bowel management, in your everyday life you’re generally very alone with this*”.

## 4. Confidence and Independence

Knowing other children with Hirschsprung’s disease is an essential part of children accepting the chronic disease and its potential implications as part of their lives. As Hirschsprung’s disease is so rare, hardly any affected children or teenagers know others in their school or town. For this reason, young patients and their siblings are encouraged to come to all SoMA e.V. meetings and conferences. Age-appropriate fun activities are scheduled for them whilst their parents join in the informative programme.

From about eight years of age, there are online fun workshops where children can get to know others with Hirschsprung’s disease whilst playing games and talking about school and their hobbies. These workshops are run by young adults who have an anorectal malformation or Hirschsprung’s disease themselves—a great encouragement for the young children to realise that “Others have (had) similar issues and it’s possible to find a good way for myself!”.

The older children become, the more important the following becomes:

- Being able to join in everyday activities with their peers;
- Finding a way of dealing with potential residual symptoms at school and in their social lives;
- Knowing other children/youths with the rare condition;
- Becoming confident and independent;
- Being able to address questions around sexuality;
- Having intimate relationships.

An important step toward these goals is for the children to learn how to perform bowel management/irrigations themselves, should they need these on a regular basis [11]. In order to achieve this, SoMA e.V. organises a four-day workshop with a paediatric surgeon and two experienced nurses who train the children from about nine years of age to perform colonic irrigation without their parents’ help [2,12,13]. Part of the programme is also to explain Hirschsprung’s disease in an appropriate way to the children, so they know their condition and can explain it to others. For parents, the workshop is an opportunity to practice letting go of their children.

Teenagers with continence issues may be reluctant to go to youth camps or join activities [9] where they are away from the safety of their home. From thirteen years of age, SoMA e.V. invites them to go on a youth camp, which is run by young adults who are affected themselves and an experienced nurse who is available for assistance if needed. For many teenagers, this is their first time away from home without their parents, which is a great boost for their confidence. Besides, in day-to-day social life and school, they often find it difficult to talk about their condition, but in the protected environment of the patient organisation’s youth camp, it is possible to open up and find mutual understanding and support.

Quotes from parents:

“*A great big step towards independence, when our child can do irrigations himself*”.

“*We’re enjoying the SoMA-community and think our child will be well connected even when she becomes independent of us*”.

A quote from an 11-year-old boy:

“*Dear SoMA-workers, it was a wonderful time with you. I learnt a lot about bowel management and my condition. The best thing was playing with my friends. Thank you!*”.

## 5. Transition and Adulthood

The necessity of the transition of care is widely recognised, but in many places, the transition is still not in place, as there are not enough clinicians who also have sufficient knowledge and experience of the paediatric condition to support adults [14,15,16]. Questions that may arise for adult patients are as follows:

- Where do I find adequate follow-up care as an adult?
- Are there any issues regarding sexual function and fertility?
- Are there any issues regarding pregnancy and birth?
- How likely is it to pass on the condition genetically?
- What is to be expected in the course of life with Hirschsprung’s disease?

Adulthood comes with many open questions regarding the long-term impact of Hirschsprung’s disease, including psychological issues. SoMA e.V. connects people with those questions and offers the possibility of exchanging thoughts and experiences regarding the often very intimate and sensitive issues. As finding medical experts as an adult patient is so difficult [15,16,17], having the possibility to exchange experiences often is the only way of accessing helpful information and support.

A quote from a patient after participating in a SoMA weekend seminar for adults:

“*I am not alone! We are all very different people with our conditions, but our problems in everyday life, job, family, relationships and free time are pretty similar. This weekend helped me, it gave me courage and strength for my future*”.

## 6. Conclusions

Hirschsprung’s disease is a rare and complex medical condition. As described, for the parents of affected children and the patients themselves, it may also come with various psychosocial challenges throughout the course of their lives. Patient organisations pursue a holistic approach: they aim to complement the medical treatment and support patients and their families in their day-to-day life. By listening, providing information and advice, and networking and sharing experiences, they promote management strategies and confidence and make a valuable contribution to ensuring a better understanding of the rare and complex condition.

According to the current European guidelines for the management of rectosigmoid Hirschsprung’s disease, part of the medical advice should be a referral of all patients and their families to patient organisations as early as possible [3].

Together with multidisciplinary medical experts, patient organisations can play an important role in improving the quality of life of patients with Hirschsprung’s disease at all ages.
